# Genome-Wide Association Study Identifies Novel Colony Stimulating Factor 1 Locus Conferring Susceptibility to Cryptococcosis in Human Immunodeficiency Virus-Infected South Africans

**DOI:** 10.1093/ofid/ofaa489

**Published:** 2020-10-16

**Authors:** Shichina Kannambath, Joseph N Jarvis, Rachel M Wake, Nicky Longley, Angela Loyse, Vicky Matzaraki, Raúl Aguirre-Gamboa, Cisca Wijmenga, Ronan Doyle, Maria Paximadis, Caroline T Tiemessen, Vinod Kumar, Alan Pittman, Graeme Meintjes, Thomas S Harrison, Mihai G Netea, Tihana Bicanic

**Affiliations:** 1 Institute of Infection and Immunity, St George’s University of London, London, United Kingdom; 2 National Institute of Health Research Biomedical Research Centre at Guy’s and St Thomas’ Hospital and King’s College London, London, United Kingdom; 3 Department of Clinical Research, Faculty of Infectious and Tropical Diseases, London School of Hygiene & Tropical Medicine, London, United Kingdom; 4 Botswana Harvard AIDS Institute Partnership, Gaborone, Botswana; 5 Clinical Academic Group in Infection, St George’s Hospital NHS Trust, London, United Kingdom; 6 University of Groningen, University Medical Center Groningen, Department of Genetics, Groningen, the Netherlands; 7 Centre for HIV and STIs, National Institute for Communicable Diseases and Faculty of Health Sciences, University of the Witwatersrand, Johannesburg, South Africa; 8 Department of Medicine and Wellcome Centre for Infectious Diseases Research in Africa, Institute of Infectious Disease and Molecular Medicine, University of Cape Town, Cape Town, South Africa; 9 Department of Internal Medicine and Radboud Center for Infectious Diseases, Radboud University, Nijmegen, the Netherlands; 10 Department for Genomics & Immunoregulation, Life and Medical Sciences Institute (LIMES), University of Bonn, Bonn, Germany

**Keywords:** Africa, *Cryptococcal meningitis*, genome-wide association study (GWAS), HIV, macrophage colony-stimulating factor (M-CSF)

## Abstract

**Background:**

*Cryptococcus* is the most common cause of meningitis in human immunodeficiency virus (HIV)-infected Africans. Despite universal exposure, only 5%–10% of patients with HIV/acquired immune deficiency syndrome and profound CD4^+^ T-cell depletion develop disseminated cryptococcosis: host genetic factors may play a role. Prior targeted immunogenetic studies in cryptococcosis have comprised few Africans.

**Methods:**

We analyzed genome-wide single-nucleotide polymorphism (SNP) genotype data from 524 patients of African descent: 243 cases (advanced HIV with cryptococcal antigenemia and/or cryptococcal meningitis) and 281 controls (advanced HIV, no history of cryptococcosis, negative serum cryptococcal antigen).

**Results:**

Six loci upstream of the colony-stimulating factor 1 (*CSF1*) gene, encoding macrophage colony-stimulating factor (M-CSF) were associated with susceptibility to cryptococcosis at *P* < 10^–6^ and remained significantly associated in a second South African cohort (83 cases; 128 controls). Meta-analysis of the genotyped *CSF1* SNP rs1999713 showed an odds ratio for cryptococcosis susceptibility of 0.53 (95% confidence interval, 0.42–0.66; *P* =* *5.96 × 10^−8^). Ex vivo functional validation and transcriptomic studies confirmed the importance of macrophage activation by M-CSF in host defence against *Cryptococcus* in HIV-infected patients and healthy, ethnically matched controls.

**Conclusions:**

This first genome-wide association study of susceptibility to cryptococcosis has identified novel and immunologically relevant susceptibility loci, which may help define novel strategies for prevention or immunotherapy of HIV-associated cryptococcal meningitis.

## Teaser

In this first GWAS conducted in cryptococcosis, we describe, replicate and functionally validate novel loci upstream of the colony stimulating factor 1 (CSF1) gene encoding macrophage colony-stimulating factor (M-CSF) as associated with susceptibility to cryptococcosis in HIV-infected South Africans.

The fungus *Cryptococcus* is a common cause of meningitis in people with human immunodeficiency virus (HIV)/acquired immune deficiency syndrome (AIDS), and it is responsible for 15% of all AIDS-related deaths globally [1]. Despite antiretroviral therapy (ART) rollout, the incidence of cryptococcal meningitis (CM) remains high in Africa and is estimated at ~200 000 cases annually [1]. In Africa, outcomes of current therapy are poor, with acute mortality of 25%–40% even with optimized therapy within a randomized multicenter trial [2] and 70% in “real-world” settings [3].

Exposure to *Cryptococcus*, an environmental saprophyte, is universal via inhalation. A population seroprevalence survey in the United States showed that anticryptococcal antibodies are common [4]. Disseminated cryptococcal infection, manifesting as meningoencephalitis, usually occurs in individuals with depressed cell-mediated immunity, typically presenting as an opportunistic infection in advanced HIV (CD4 T-cell count <100/µL). Despite likely exposure, not all patients with advanced HIV develop disseminated cryptococcosis: prevalence of cryptococcal antigenemia (CRAG), representing early dissemination from the lungs, is approximately 6% in this population [1]. After treatment of both cryptococcosis and underlying HIV, despite comparable CD4 counts, CRAG-positive individuals have a 12-month mortality rate approximately 3 times greater than CRAG-negative controls [5], suggesting that additional host immune factors, beyond that reflected by the CD4 count, may contribute to cryptococcosis susceptibility.

Host immunity to *Cryptococcus neoformans*, an intracellular pathogen, requires coordinated innate and adaptive responses, with phagocytosis by classically activated (M1) macrophages promoting robust Th1-type responses and the production of proinflammatory cytokines (tumor necrosis factor [TNF]-α and interferon [IFN]-γ) playing a central role in fungal clearance and host survival [3, 6]. In apparently immunocompetent hosts, several CM susceptibility determinants have been described, including idiopathic CD4 lymphopenia, antibodies to granulocyte-macrophage colony-stimulating factor (CSF) and IFN-γ and Fc-γ receptor, and mannose-binding lectin polymorphisms [3, 7, 8].

Prior immunogenetic studies performed in CM have studied candidate genes in small populations (n = 100–150) comprising few African individuals [3, 7–9]. In the only CM genetic susceptibility study in HIV-positive patients, targeted sequencing of the Fc-γ receptor in a cohort of 164 predominantly Caucasian men (55 HIV-positive with CM; 54 HIV-positive and 55 HIV-negative controls without CM) demonstrated that individuals homozygous for the Fc-γR3A 158V polymorphism had 20-fold increased odds of developing CM [9]. Despite sub-Saharan Africa having a high infectious disease burden, few genome-wide association studies (GWAS) of infectious disease susceptibility have been conducted in people of African descent: published studies include tuberculosis [10] and malaria [11, 12]. Specific challenges to GWAS in the African population include higher genetic diversity, low linkage disequilibrium, and more complex genetic structure [13], although, in the long-term, these aspects can be exploited for fine mapping of association signals.

In this study, we report on the first GWAS of genetic susceptibility to cryptococcosis in an HIV-infected population, using deoxyribonucleic acid from a discovery cohort of 524 cases and controls of African descent recruited in Cape Town 2005–2014 and a validation cohort of 211 recruited in Johannesburg 2015–2017.

## METHODS

### Human Cohorts

#### Discovery and Validation Cohort

For the discovery cohort, 243 cases were recruited as part of 4 clinical trials (1 observational, 3 randomized) of HIV-associated CM and a CRAG study in ART-naive adults conducted in Cape Town, South Africa 2005–2014 [14–18]. Cases had disseminated cryptococcal infection and/or CM as confirmed by positive serum and/or CSF cryptococcal antigen and/or CSF culture. Two hundred eighty-one controls were recruited contemporaneously at the same hospital and referring clinic as the cases and had no history of cryptococcal disease and a negative serum cryptococcal antigen. All cases and controls were HIV-positive adults (age ≥18) with nadir CD4 cell count <100/μL who were ART-naive or within 3 months of starting ART. The validation cohort included 63 cases and 128 controls with CD4 cell count <100/μL recruited as part of a cryptococcal antigen screening study in ART-naive HIV-infected adults in 2015–2017 [19] (Table 1). Twenty cases from a clinical trial of HIV-CM in Kwazulu-Natal were also included in this cohort [16].

**Table 1.** Age, Sex, and CD4 Count for Cases and Controls in Discovery and Validation Cohorts^a^

Discovery Cohort	Controls	Cases
n	218	243
Age	33 (18–66)	33 (18–62)
Sex (%F)	66%	61%
CD4 (cell/μL)	46 (23–78)	37 (16–67)
Validation Cohort		
n	128	83
Age	40 (18–76)	39 (21–68)
Sex (%F)	56%	54%
CD4 (cell/μL)	44 (1–99)	25 (1–90)

^a^Median (range) shown for continuous variables.

#### Cryptococus-Specific Transcriptome and Functional Characterization Cohort

 Ribonucleic acid sequencing (RNA-seq) was performed on peripheral blood mononuclear cells (PBMCs) from healthy volunteers of self-identified Xhosa ethnicity recruited in Cape Town. The functional characterization cohort included 5 HIV-infected patients of diverse ethnicities recruited at St George’s Hospital, London, with CD4 count <200 cells/μL and not on ART within ≤12 months. Healthy donor PBMCs used were obtained from leukocyte cones. Further details of experimental methods and computational analyses are provided in the Supplementary Methods.

#### Patient Consent Statement

The studies were approved by ethics committees at the University of Cape Town, the University of Witswatersrand, and the London School of Hygiene of Tropical Medicine. All participants gave written informed consent. 

### Genotyping and Association Analyses

Five hundred twenty-four cases and controls from the discovery cohort were genotyped using the Illumina HumanOmniExpressExome-8 v1.0 single-nucleotide polymorphism (SNP) chip, an exome-based array with >700 000 genome-wide markers and >240 000 exonic markers. Two hundred eleven samples from the validation cohort were genotyped on the Illumina GSA beadchip GSA MD v1. Samples with a low call rate (≤99%) and variants with a Hardy-Weinberg equilibrium ≤0.00001, call rate <0.99, missingness test (GENO > 0.01), and minor allele frequency (MAF) <0.001 were excluded from further analyses. Eleven genetically divergent samples were excluded from the discovery cohort and 6 from the validation cohort. A total of 245 091 variants from 513 discovery samples passed quality control and were analyzed. Variants were aligned to the 1000 Genome reference and the data were imputed using the Michigan Imputation server. Postimputation quality controls were used to remove low-quality (r2 ≤ 0.8) imputed variants before further analyses.

The association analysis was performed, and genetic susceptibility to disseminated cryptococcosis was tested using logistic regression. *P* value distribution was assessed using a Quantile-Quantile (Q-Q) plot, and there was no inflation effect on the association analysis. Discovery and validation cohort-imputed datasets were subsequently merged, and a combined cohort association analysis was performed on 2 686 126 variants, with the significance threshold set at *P* < 5 × 10^−6^. The impact of top SNPs on gene expression was explored using eQTL information from the HaploReg and Genotype Tissue Expression (GTex) databases (see Supplementary Methods). Information on SNP association with annotated genes and variants within 500 kb of each SNP was collated. Genes associated with SNPs with *P* < 5 × 10^−3^ were included in pathway enrichment and gene ontology analyses. At the *CSF1* locus, SNP rs1999713 was hard-called on both genotyping platforms for both cohorts, so we performed a meta-analysis of the discovery and validation cohorts to negate any uncertainty from imputation, using an allele and fixed-effects model as the effect size, and direction was very similar in both the discovery and replication cohorts.

### Macrophage Colony-Stimulating Factor Functional Characterization Experiments

The PBMCs from HIV-infected patients (n = 5) and healthy volunteers were pretreated with macrophage-CSF (M-CSF) or anti-M-CSF antibody and cocultured with *C neoformans* H99 (serotype A reference strain) for 24 hours. Cells were lysed, plated onto fresh SAB agar for 48 hours, and colony-forming units were counted. For the phagocytosis assays, PBMCs were pretreated as described above and then challenged with prelabeled heat-killed *C neoformans* for 24 hours at 37°C. Cells were then captured on a flow cytometer, and the percentage of cells with internalized cryptococcus were identified.

### RNA Sequencing and Analyses

The PBMCs were stimulated with heat-killed *C neoformans* (multiplicity of infection = 0.1) for 24 hours. Ribonucleic acid was extracted, and a sequencing library was prepared and sequenced as described in Supplementary Methods. After quality-control measures, reads were mapped to the human reference genome (hg19). Reads were annotated and differentially expressed genes between controls and Cn-treated samples were identified. Genes with significant differential expression were used in gene ontology and pathway analyses.

### Availability of Data and Materials

The human SNP array summary datasets and raw RNA-seq data supporting the conclusions of this article are available on figshare via link https://figshare.com/s/b953f3192c77cef0be98. The software and detailed analyses steps we undertook are detailed via link https://github.com/alanmichaelpittman100/Crypto-GWAS.

## RESULTS

### Genome-Wide Association Analysis

We performed a GWAS of *Cryptococcus* susceptibility in a discovery cohort of 524 age-, gender-, and CD4 count-matched South African HIV-infected patients: cases with disseminated cryptococcosis (defined as positive serum CRAG and/or CM, n = 243) and controls (n = 281) with no cryptococcosis. The validation cohort comprised 83 cases and 128 controls of African descent (Table 1). After imputation and quality-control measures (Supplementary Figure 1a), ~9.2 million variants from 240 cases and 273 controls (discovery) and 79 cases and 126 controls (validation) were analyzed using regression analysis.

**Figure 1.**
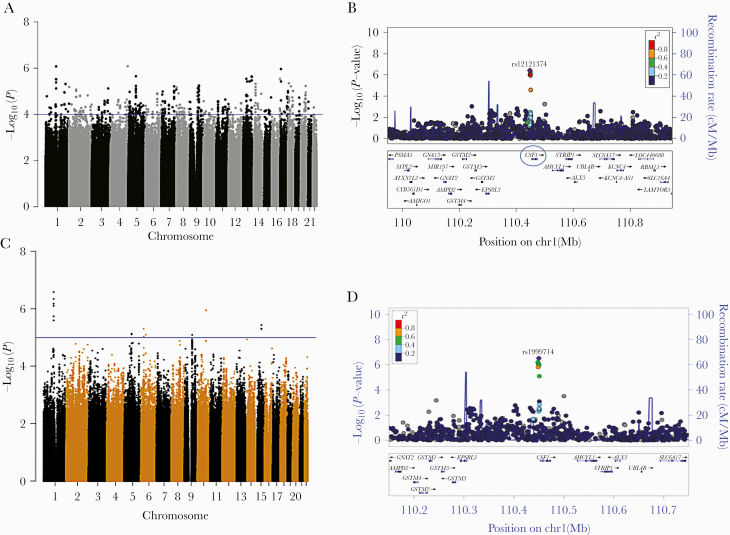
Manhattan plots and regional association plots for Discovery (A,B) and Combined (C,D) cohort genome-wide association study. (A) Manhattan plot showing the genome-wide *P* values of association with cryptococcal meningitis in the Discovery cohort. The y-axis represents the log_10_*P* values of single-nucleotide polymorphisms (SNPs), and their chromosomal positions are shown on the x-axis. The horizontal blue line shows the significance threshold of *P* < 1 × 10^−4^. *P* values were obtained by logistic regression. Six SNPs upstream of the *CSF1* gene on chr1 lay above this threshold, including a SNP at the enhancer region of *CSF1*. (B) Regional association plots at the Chr1 associated with *CSF1* genes. Estimated recombination rates are shown in blue to reflect the local linkage disequilibrium structure around the associated top SNP and its correlated proxies, with bright red indicating highly correlated and pale red indicating weakly correlated. (C) Manhattan plot showing the genome-wide *P* values of association with cryptococcosis in the Combined cohort. The horizontal blue line shows the significance threshold of *P* < 1 × 10^−5^. The *P* values were obtained through linear models (lrt) in GEMMA software with 15 ancestry principal components as covariates. (D) Regional association plots at the Chr1 *CSF1* gene locus.

In the discovery cohort, we identified multiple loci associated with susceptibility to cryptococcosis (Figure 1a). Although no individual SNP passed the genome-wide significance threshold *P* < 5 × 10^–8^, we identified 49 SNPs with *P* < 10^–5^ associated with cryptococcosis (Table 2). Six of the top susceptibility SNPs (*P* < 7.54 × 10^–6^; odds ratio [OR] = 0.49–0.53) were located within 2.5 kb upstream of the *CSF1* gene encoding M-CSF (Figure 1b), a cytokine promoting macrophage activation and phagocytosis. The top associated SNP rs1999714 (OR = 0.49; *P* = 8.39 × 10^–7^) was located in the block of linkage disequilibrium (LD) of ~2.5 kb, defined by significant r^2^ >0.5 LD of surrounding SNPs with rs1999714) close to the *CSF1* gene (Figure 1b). Another top variant, rs12124202 (OR = 0.53; *P* = 7.54 × 10^–6^), was in the gene enhancer region (position GRCh38.p12 chr1: 109 905 601–109 906 901, GeneHancer ID GH01J109905), and other SNPs (including rs1999714) were all close to the *CSF1* regulatory region. However, exploring the impact of these candidate SNPs on gene on gene regulation using a number of databases (Supplementary Methods) revealed no expression quantitative traits for any of the CSF1 SNPs, including the SNP in the enhancer region of CSF1. Other susceptibility SNPs of potential relevance to *Cryptococcus*-macrophage interactions included rs6768912 (OR = 1.8; *P* = 7.56 × 10^–6^) in the intronic region of *NCEH1* (neutral cholesterol ester hydrolase) and rs7213159 (OR = 1.9; *P* = 9.79 × 10^–6^), a noncoding transcript variant of *CSNK1D* (casein kinase I). NCEH1 encodes neutral cholesterol ester hydrolase, an enzyme-removing cholesterol, which plays a pivotal role in antiviral responses (including to HIV), in macrophages [20]. Gene silencing of the *CSNK1D* gene has been shown to significantly reduce intracellular mycobacterial load in murine macrophages [21] (Table 2).

**Table 2.** List of Variants (*P* < 1.0 × 10^−5^) Associated With Cryptococcosis in Discovery Cohort

CHR	BP	SNP	Closest Gene	Gene Region	Minor/Major	Frequency Cases/Control	*P* Value	OR
1	110450033	rs1999714	CSF1	Upstream gene variant	T/G	0.21/0.35	8.4E-07	0.50
	110448080	rs12121374	CSF1	Upstream gene variant	C/T	0.23/0.36	3E-06	0.52
	110449962	rs1999715	CSF1	Upstream gene variant	A/C	0.24/0.37	3E-06	0.53
	110450177	rs1999713	CSF1	Upstream gene variant	C/T	0.24/0.37	4.1E-06	0.53
	110448590	rs12124202	CSF1	Enhancer	A/G	0.23/0.35	7.5E-06	0.53
	210048819	rs2064163	DIEXF	Upstream gene variant	G/T	0.28/0.42	4.8E-06	0.55
2	788370	rs4854383	AC113607.1	Intronic	G/C	0.32/0.20	6.5E-06	1.92
	74452327	rs12476235	RP11-287D1.3	Intronic	A/G	0.26/0.15	8.4E-06	2.03
	74454448	rs60003281	RP11-287D1.3	Intronic	C/G	0.26/0.15	9.7E-06	2.01
3	172378536	rs6768912	NCEH1	Intronic	A/C	0.5/0.36	7.6E-06	1.78
4	182214247	rs6846320	RP11-665C14.2	Upstream gene variant	A/C	0.21/0.10	8.2E-07	2.40
5	78878938	rs12514204	PAPD4	Upstream gene variant	C/G	0.51/0.36	2.2E-06	1.83
	78881151	rs72635607	PAPD4	Upstream gene variant	T/C	0.17/0.29	5.9E-06	0.50
	78896859	rs72635609	PAPD4	Upstream gene variant	T/G	0.17/0.29	7.5E-06	0.51
	78064511	rs10079201	LHFPL2	Upstream gene variant	A/G	0.16/0.28	9.1E-06	0.51
7	133876985	rs2068375	LRGUK	Intronic	T/C	0.03/0.10	6.1E-06	0.28
	157726548	rs111508983	PTPRN2	Intronic	G/A	0.12/0.04	8.4E-06	3.00
	133885512	rs4732006	LRGUK	Intronic	G/A	0.03/0.10	9.8E-06	0.29
	133888726	rs78496580	LRGUK	Intronic	A/G	0.03/0.10	9.8E-06	0.29
	133888979	rs79956644	LRGUK	Intronic	A/C	0.03/0.10	9.8E-06	0.29
	133891059	rs76591747	LRGUK	Intronic	T/G	0.03/0.10	9.8E-06	0.29
	133895592	rs77103757	LRGUK	Intronic	T/C	0.03/0.10	9.8E-06	0.29
8	567740	rs1703893	ERICH1	Upstream gene variant	G/A	0.12/0.22	6.7E-06	0.46
9	92263074	rs78649414	GADD45G	intronic	C/G	0.07/0.16	5.7E-06	0.39
	92258429	rs7025202	GADD45G	intronic	G/A	0.10/0.20	6.3E-06	0.44
	80978737	rs73651328	PSAT1	Upstream gene variant	G/A	0.06/0.15	7.3E-06	0.38
	92263407	rs74398964	GADD45G	Intronic	T/C	0.07/0.16	8.4E-06	0.40
	92261102	rs80245985	GADD45G	Intronic	T/C	0.08/0.17	9.9E-06	0.42
13	108504208	rs1396593	FAM155A	Intronic	A/G	0.10/0.03	2.3E-06	3.70
	108505141	rs9520606	FAM155A	Intronic	T/A	0.10/0.03	2.3E-06	3.70
	108506375	rs2136266	FAM155A	Intronic	T/C	0.10/0.03	2.3E-06	3.70
	51950848	rs79789954	INTS6	Intronic	T/C	0.08/0.17	2.9E-06	0.40
	108503869	rs9520603	FAM155A	Intronic	A/G	0.10/0.03	3.3E-06	3.48
	108503995	rs9520605	FAM155A	Intronic	C/T	0.12/0.04	3.4E-06	3.12
	85474990	rs9602571	RP11-531P20.1	Upstream gene variant	A/G	0.09/0.19	3.8E-06	0.42
	85475371	rs9602572	RP11-531P20.1	Upstream gene variant	G/C	0.09/0.19	3.8E-06	0.42
	60084350	rs187657736	RNU7-88P	Upstream gene variant	T/G	0.10/0.03	3.8E-06	3.62
14	34930846	rs74046057	SPTSSA	Intronic	T/C	0.53/0.39	4.8E-06	1.78
	34930523	rs57186368	SPTSSA	Intronic	T/C	0.53/0.39	6.5E-06	1.77
	34928860	rs12434081	SPTSSA	Intronic	G/A	0.53/0.39	8.7E-06	1.76
16	85146454	rs75842988	FAM92B	Upstream gene variant	A/G	0.24/0.12	2.9E-06	2.20
17	5568721	rs115470097	NLRP1	Upstream gene variant	G/A	0.18/0.07	1.1E-06	2.64
	5568733	rs111541610	NLRP1	Upstream gene variant	C/T	0.19/0.09	3.7E-06	2.37
	80223048	rs7213159	CSNK1D	Intronic	C/T	0.32/0.20	9.8E-06	1.89
18	8211568	rs112514564	PTPRM	Intronic	C/T	0.11/0.03	4.5E-06	3.35
	29586237	rs12454708	RNF125	Upstream gene variant	C/G	0.03/0.10	6.1E-06	0.28
	52320409	rs11877451	C18orf26	Upstream gene variant	G/A	0.17/0.28	9.7E-06	0.51
	52322820	rs7233418	C18orf26	Upstream gene variant	G/C	0.17/0.28	9.7E-06	0.51
20	49810845	rs78757036	AL035457.1	Upstream gene variant	A/G	0.08/0.17	5.9E-06	0.41

Abbreviations: BP, base pair; CHR, chromosome; OR, odds ratio; SNP, single-nucleotide polymorphism.

**Figure 2.**
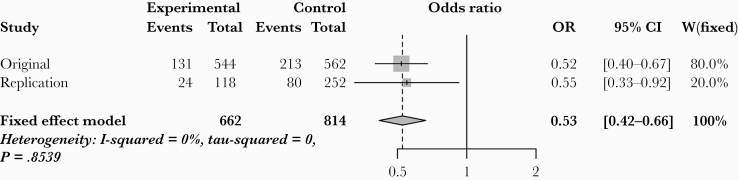
Meta-analysis and forest plot of hard-called genotyped *CSF1* single-nucleotide polymorphism rs1999713, present in both discovery and validation cohorts. Model shown is allele test under a fixed-effects model (heterogeneity, I^2^ = 0%, *P* = .8539). The presence of rs1999713 was associated with an odds ratio (OR) of 0.53 (95% confidence interval [CI], 0.42–0.66; *P *=* *5.96 × 10^−8^) for development of cryptococcosis in the combined cohort.

To validate findings from our discovery cohort, we performed GWAS in a separate South African cohort of 79 cases and 126 controls. The *CSF1* SNPs were independently significant in this smaller cohort (OR* *=* *0.52–0.63; *P* < .05) (Table 3). In the combined cohort of 319 cases and 399 controls, all 6 *CSF1* SNPs remained significantly associated with cryptococcosis susceptibility (Table 3, Figure 1c and d, Supplementary Figure 2). A meta-analysis of the (nonimputed) genotyped *CSF1* SNP rs1999713 (present in both discovery and validation cohorts) using a fixed-effects allele model generated an OR of 0.53 (95% confidence interval [CI], 0.42–0.66, *P *=* *5.96 × 10^–8^; heterogeneity, I^2^ = 0%, *P* = .8539) in the combined cohort (Figure 2).

**Table 3.** List of Variants (*P* < 1.0 × 10^−5^) Associated With Cryptococcosis in Combined GWAS (Discovery and Validation) Cohort

Combined Cohort									Discovery Cohort			Replication Series		
CHR	BP	SNP	Closest Gene	Gene Region	Minor/ Major	Frequency Case/ Control	*P* Value	OR	Frequency Case/ Control	*P* Value	OR	Frequency Case/ Control	*P* Value	OR
1	110450033	rs1999714	CSF1	Upstream gene variant	T/G	0.2401/0.3219	2.62E-07	0.6656	0.2104/0.3553	3.112E-07	0.4835	0.178/0.2817	.03136	0.5519
1	110449962	rs1999715	CSF1	Upstream gene variant	A/C	0.2616/0.3342	4.55E-07	0.7059	0.2333/0.3755	8.836E-07	0.5063	0.1949/0.3175	.01424	0.5205
1	110451118	rs7535558	CSF1	Upstream gene variant	C/T	0.3146/0.3808	6.66E-07	0.7461	0.2958/0.4304	8.153E-06	0.556	0.2627/0.381	.02558	0.579
1	110450177	rs1999713	CSF1	Upstream gene variant	C/T	0.2649/0.3354	7.90E-07	0.7141	0.2333/0.3736	1.193E-06	0.5102	0.2119/0.3175	.03575	0.578
10	91937740	rs4933565	LINC01375	Upstream gene variant	T/G	0.2715/0.1855	1.11E-06	1.637	n/a	n/a	n/a	0.3305/0.2183	.0208	1.768
10	91937734	rs4933564	LINC01375	Upstream gene variant	T/A	0.2715/0.1855	1.14E-06	1.637	n/a	n/a	n/a	0.3305/0.2183	.0208	1.768
1	110448590	rs12124202	CSF1	Enhancer	A/G	0.2500/0.3256	1.83E-06	0.6906	0.2188/0.3553	1.563E-06	0.508	0.1949/0.2897	.0526	0.5937
1	110448080	rs12121374	CSF1	Upstream gene variant	T/C	0.2566/0.3256	2.54E-06	0.7152	0.2188/0.3608	6.303E-07	0.496	0.2119/0.2976	.08344	0.6344
15	68182254	rs28445794	RNU6-1	Upstream gene variant	C/T	0.1887/0.2002	3.69E-06	0.9292	0.1229/0.2216	3.364E-05	0.4922	0.1949/0.2778	.08681	0.6295
15	68180746	rs34743389	RNU6-1	Upstream gene variant	A/G	0.1904/0.2064	4.90E-06	0.9043	0.1271/0.2253	0.000043	0.5007	0.1949/0.2817	.07376	0.6172
6	29833057	rs3128900	HLA-H	intronic	T/G	0.1755/0.1806	4.97E-06	0.9658	0.2417/0.1557	0.0005349	1.728	0.1525/0.131	.5745	1.195
15	68180471	rs62014301	RNU6-1	Upstream gene variant	A/G	0.1904/0.2064	5.05E-06	0.9043	0.1271/0.2253	0.000043	0.5007	0.1949/0.2817	.07376	0.6172
5	78638719	rs114228467	JMY, HOMER1	Upstream gene variant	A/G	0.0464/0.0147	7.39E-06	3.249	n/a	n/a	n/a	0.0593/0.0079	.002787	7.883
5	78635829	rs148260321	JMY, HOMER1	Upstream gene variant	G/C	0.0464/0.0147	7.78E-06	3.249	n/a	n/a	n/a	0.0593/0.0079	.002787	7.883
6	52162415	rs61126502	MCM3, IL17F	Upstream gene variant	T/C	0.0431/0.0405	8.06E-06	1.065	n/a	n/a	n/a	0.0254/0.0952	.01611	0.2478
9	81835737	rs273465	LOC101927450	Upstream gene variant	A/C	0.154/0.1671	8.13E-06	0.9073	0.1042/0.2015	1.817E-05	0.4609	0.1356/0.1905	.1933	0.6667
6	29943688	rs2394251	HLA-H	intronic	C/G	0.2566/0.317	9.32E-06	0.7439	n/a	n/a	n/a	0.2712/0.2063	.1653	1.431

Abbreviations: BP, ; CHR, ; GWAS, genome-wide associated study; n/a, not applicable; OR, odds ratio; SNP, single-nucleotide polymorphisms.

### Transcriptomics in Healthy Peripheral Blood Mononuclear Cells and Overlap With Genome-Wide Association Study Findings

Using PBMCs from 6 healthy donors of self-identified Xhosa ethnicity, we performed RNA-seq after stimulation with heat-killed *C neoformans* for 24 hours. Compared with unstimulated PBMCs, 653 genes were significantly up- or down-regulated (fold change >2; adjusted value <0.05) (Supplementary Table 1). CSF1 was significantly up-regulated (log_2_-fold change 2.55, adjusted *P* = 2.6 × 10^–16^) along with IFN-γ, TNFα, CCL1, and CCL8 (Supplementary Table 1). Looking for an overlap between genes differentially expressed in the RNA-seq experiment and genes associated with significant SNPs (*P* < 1 × 10^–3^) in the GWAS, we found 38 common genes (Table 4), 9 of which, including CSF1, were significantly up-regulated upon cryptococcal stimulation. Genes common to GWAS and RNA-seq were associated with functions such as cell adhesion (*CD36*, *CSF1*, *NRG1*, and *TGFBI*), macrophage differentiation (*CSF1*, *IL31RA*), cell proliferation (*RASGRF1*, *CSF1*, *NRG1*, *SPOCK1*, and *TGFBI*), and ion transport (*ATP6V0D2*, *CACNA2D3*, *CTTNBP2*, *KCNJ6*, *SLC8A1*, and *SLCO2B1*).

**Table 4.** List of GWAS-Identified Genes (Variants With *P* < .001) Showing Differential Expression in the RNA-seq Experiment (Differential Log_2_ Fold Change ≥1)

Common Genes	Number of Variants (*P* < 1.0 × 10^−3^)	Log_2_ Fold Change	padj
IL31RA	3	3.65	2.5E-26
CSF1	8	2.55	2.6E-16
BCL2L14	2	1.90	3.5E-08
CCL24	1	1.59	0.00242
DPF3	13	1.06	0.02754
SAMD4A	7	1.48	2.8E-05
NDRG2	1	1.41	8.8E-06
HPSE2	2	1.27	0.04782
RASGRF1	1	1.18	0.00489
CD36	2	−1.01	0.03026
C10orf54	2	−1.02	0.00183
NAV1	1	−1.05	0.01309
NAV2	49	−1.06	0.01309
GPR141	1	−1.11	0.02783
INSR	1	−1.21	0.0072
MUC16	1	−1.21	0.0015
HRASLS5	4	−1.27	0.03434
PCSK5	6	−1.27	0.03238
ABCA13	9	−1.28	0.00193
SLC47A1	1	−1.34	0.04405
PXDN	4	−1.35	0.0147
EEPD1	1	−1.40	0.00358
NHSL1	1	−1.43	0.00021
ATP6V0D2	1	−1.46	0.00209
SLC8A1	3	−1.47	0.01127
SPOCK1	2	−1.51	0.00183
EPB41L3	1	−1.54	0.01091
KCNJ6	1	−1.61	6.6E-10
SLCO2B1	1	−1.69	0.00552
NRG1	2	−1.74	0.0002
CTTNBP2	3	−1.82	0.00173
TGFBI	1	−1.97	0.00059
GLIS3	1	−2.03	6E-06
CACNA2D3	1	−2.08	3.6E-06
NCEH1	3	−2.11	6.5E-05
DLEU7	2	−2.20	1E-08
LTBP2	1	−2.47	4.2E-09
PID1	4	−3.06	1.1E-08

Abbreviations: CSF1, colony-stimulating factor 1; GWAS, genome-wide associated study; padj, adjusted P value; RNA-seq, ribonucleic acid sequence.

^a^The top 9 genes, including CSF1, were significantly up-regulated in response to cryptococcal stimulation of peripheral blood mononuclear cells from healthy Xhosa volunteers.

Gene ontology analysis of differentially expressed genes in healthy controls identified enrichment of cytokine activity, phagocytosis, complement, and T-cell proliferation (Supplementary Table 2). Pathway analysis of these genes identified enrichment of cytokine-cytokine receptor interaction, complement and coagulation cascades, and Toll-like signaling pathways (Supplementary Table 2). These findings lend further support to the importance of genes involving macrophage activation, differentiation, and phagocytosis, including CSF1, to cryptococcal immune responses in the South African population.

### Functional Characterization in Peripheral Blood Mononuclear Cells From Patients With Advanced Human Immunodeficiency Virus

**Figure 3.**
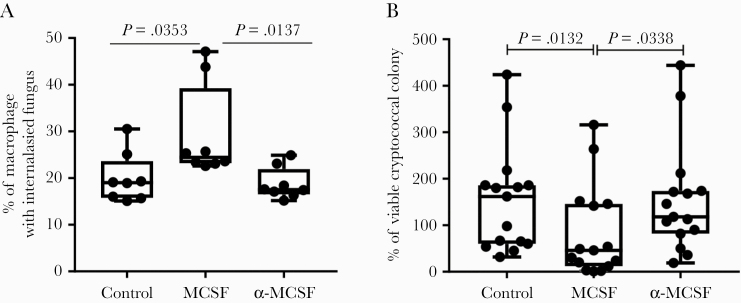
*Cryptococcus* internalization and killing by peripheral blood mononuclear cells (PBMCs) from patients with advanced human immunodeficiency virus (HIV) infection (*n *=* *5). The PBMCs were pretreated to block macrophage colony-stimulating factor (MCSF) receptors using α-MCSF or provided with additional MCSF and then coinfected with heat-killed cryptococcus. (a) The PBMCs from HIV-infected patients showed significantly higher internalization of *Cryptococcus* when treated with additional MCSF. (b) Human immunodeficiency virus-infected patient PBMCs also exhibit better killing of *Cryptococcus* compared with the nontreated PBMCs. Phagocytosis and fungal killing in anti-MCSF-treated samples were similar to controls, suggesting incomplete receptor block or lack of endogenous MCSF production in patients. For the 5 patients, there were 2 technical replicates for the phagocytosis experiments and 3 for the fungal killing experiments: all data points are shown on the graph. *P* values are shown using 2-sided *t* test; box and whiskers plot shows median ± interquartile range.

To further examine the importance of M-CSF in cryptococcal phagocytosis and killing, we performed ex vivo experiments using PBMCs of 5 HIV-infected patients (ART-naive, CD4 count <200 cells/μL). Exogenous M-CSF significantly improved cryptococcal phagocytosis and killing by HIV-infected PBMCs (Figure 3). When M-CSF receptors were blocked with specific antibodies, phagocytosis and fungal killing were similar to that of unstimulated PBMCs, suggesting either incomplete receptor block or absence of endogenous M-CSF production in patients (Figure 3).

## DISCUSSION

Despite bearing the largest infectious disease burden, African individuals are underrepresented in studies of disease susceptibility [22]. Globally, fungal infections pose a major threat to human health as a result of the expansion of immunosuppressive interventions and the ongoing HIV epidemic [23]. Due to the challenges in recruiting large enough cohorts, the first GWAS in an invasive fungal infection (candidaemia) was published in 2014 [24]. The present study is the first to be conducted for cryptococcosis, taking 12 years (2005–2017) to enroll a total of 735 patients.

Unlike prior targeted sequencing approaches, we took an unbiased, hypothesis-generating approach as used previously for candidemia [24, 25], combining GWAS in a clearly defined case-control cohort, backed up by validation in a second cohort, transcriptomics in ethnically matched healthy controls and functional studies. Although no individual locus reached genome-wide significance, meta-analysis of the nonimputed genotyped *CSF1* SNP rs1999713 demonstrated *P* < 10^–8^ (OR* *= 0.53; 95% CI, 0.42–0.66; *P *= 5.96 × 10^–8^) and was independently significant in both our discovery and validation cohorts. It is worth noting that this result was obtained in an African population in which GWAS power was limited by extensive genetic diversity and low linkage disequilibrium [13].

Although no SNPs identified lay within coding regions, we identified immunologically plausible upstream genetic variants with potential regulatory roles, notably 5 SNPs in the regulatory region and 1 SNP on the enhancer region of the *CSF1* gene encoding M-CSF. Macrophage-CSF induces survival, proliferation, chemotaxis, differentiation, and activation of monocytes/macrophages, including microglia [26, 27]. All 6 SNPs were confirmed in the validation cohort, remaining significantly associated with risk of cryptococcosis in the combined cohort. Although we did not have CSF1 genotype data for the healthy controls to link with gene expression, *CSF1* was also one of the most highly up-regulated genes upon cryptococcal stimulation of PBMCs from healthy, ethnically matched volunteers, and experiments confirmed the importance of M-CSF in uptake and killing of *Cryptococcus* by PBMCs from HIV-infected patients.

Exogenous M-CSF enhances the anticryptococcal activity of human monocyte-derived macrophages and enhanced cryptococcal killing in a murine model, and it was synergistic with fluconazole [28–30]. Macrophage-CSF is one of the principal regulators of macrophage function [27, 31], acting as a potent proliferation signal, increasing blood and tissue macrophage numbers [31–33]. Macrophage-CSF-primed macrophages are typically more phagocytic and less competent at antigen presentation, primed to M2 stimuli [32]; however, M-CSF does not induce a full M2 phenotype, with M-CSF-primed macrophages able to respond to a variety of proinflammatory stimuli including IFN-γ and Toll-like receptor activation [31, 32, 34, 35]. Macrophage-CSF acts synergistically with IFN-γ to drive proinflammatory chemokine production including CCL2 (MCP-1) [31], and it is expressed in a subset of T-cells that also express Th1 markers [36]. T-cell derived M-CSF has been shown to play a crucial role in the control of bloodborne intracellular pathogens [36], and blocking M-CSF increases susceptibility to intracellular infections with *Listeria* and *Mycobacterium tuberculosis* [37, 38]. The exact role of M-CSF in protective anticryptococcal immune responses in the context of HIV coinfection is unclear, although extensive data demonstrating the importance of effective alveolar macrophage responses in controlling early cryptococcal infection [6], and the key role of circulating and tissue macrophage/microglial responses during later disseminated disease [39, 40], provide a plausible basis for why variations in *CSF1* gene expression might impact susceptibility to cryptococcal disease. Of interest, the genotyped *CSF1* SNP rs1999713 is common in different populations, with sampled African populations having the lowest MAF at 0.31 (comparable to 0.34 found in our control group) and East Asian populations having the highest MAF at 0.68 (https://gnomad.broadinstitute.org/).

Searching for inherited immune defects in anticryptococcal responses in the context of profound acquired CD4 T-cell depletion might seem paradoxical: yet given only a minority of patients with HIV/AIDS develop disseminated cryptococcosis despite presumed ubiquitous exposure, such an approach has the potential to highlight the contribution of other factors, including the central role of macrophage phagocytosis and killing [41]. Macrophages are also infected by HIV and act as its tissue reservoir [42, 43] and are involved in trafficking both pathogens to the central nervous system (CNS). We postulate that, in the setting of HIV-cryptococcal coinfection, genotypes rendering macrophages more permissive to uptake and intracellular survival of intracellular pathogens are likely to confer susceptibility to disseminated cryptococcosis, either through direct effects on cryptococcal intracellular burden or indirectly through an impact on HIV burden [44]. FcγR polymorphisms identified in prior targeted sequencing studies [8, 9] could exert an impact through either increasing phagocyte cargo (via increased binding and uptake of *C neoformans*-immune complexes), shown to be associated with CSF fungal burden in HIV-CM [41], and/or increased immune activation via antibody-dependent cellular cytotoxicity, leading to disruption of the blood-brain barrier or CNS tissue injury [9]. Both M-CSF and the M-CSF receptor have been proposed as targets in the treatment of HIV neurodegenerative disease [45, 46], and M-CSF treatments for invasive fungal infections have been investigated in animal models [47, 48] and early stage clinical trials [49].

Our study had several limitations. The relatively small sample size limited our statistical power, and genotype arrays differed for the 2 cohorts. The discovery cohort was genotyped on a chip biased towards European populations, whereas the validation cohort was typed using the newly available global screening array ([GSA] containing multiethnic genome-wide content), making imputation crucial for analysis of the combined cohort. Better designed genotyping chips representing African genetic diversity (such as the GSA and newer arrays under development) will mean less reliance on imputation methods to fill in the gaps in the African genomes. We lacked genotype data on the healthy volunteers that would have allowed us to examine effects of CSF1 genotype on cytokine expression upon cryptococcal stimulation. Furthermore, there was a paucity of eQTL data from African populations on the impact of the upstream variants identified on CSF1 gene expression and M-CSF production: this could be explored in future studies using PBMCs of genotyped individuals. Beyond host genotype, other unaccounted-for factors, such as those associated with environmental cryptococcal exposure, or concurrent opportunistic infections, may have an impact on cryptococcosis susceptibility.

In any GWAS of infectious disease susceptibility, pathogen variation is an additional and usually unaccounted-for element [13]. The completion of large, multisite, African phase III trials in HIV-associated CM provides the opportunity to undertake a larger pan-African GWAS of disease severity and treatment response, developing bioinformatic approaches to integrate host and pathogen genomics with host CSF immune profiling and pathogen virulence phenotyping to determine host and pathogen factors underlying poor clinical outcome [2, 50].

## CONCLUSIONS

In summary, we have identified and replicated a novel cryptococcosis susceptibility factor in HIV-infected Africans, the importance of which was further confirmed through ex vivo functional immune studies in patients with advanced HIV as well as healthy, ethnically matched controls. Our findings demonstrate that small but well defined GWAS can identify novel and immunologically relevant susceptibility loci for an important cause of mortality in an African population, provided they are replicated and complemented by functional approaches. Identifying a high-risk genotype helps elucidate disease mechanism and has the potential to identify novel strategies for targeted prevention and host-directed immunotherapy.

## Supplementary Data

Supplementary materials are available at *Open Forum Infectious Diseases* online. Consisting of data provided by the authors to benefit the reader, the posted materials are not copyedited and are the sole responsibility of the authors, so questions or comments should be addressed to the corresponding author.

ofaa489_suppl_Supplementary_Data

ofaa489_suppl_Supplementary_Materials
