# Supplementary material for: Genome-Wide Association Study Identifies Novel Colony Stimulating Factor 1 Locus Conferring Susceptibility to Cryptococcosis in Human Immunodeficiency Virus-Infected South Africans
Source: Open Forum Infect Dis. 2020 Oct 16;7(11):ofaa489. doi: 10.1093/ofid/ofaa489 (PMC7686661; doi:10.1093/ofid/ofaa489)
Supplement: ofaa489_suppl_Supplementary_Data [file ofaa489_suppl_supplementary_data.docx]

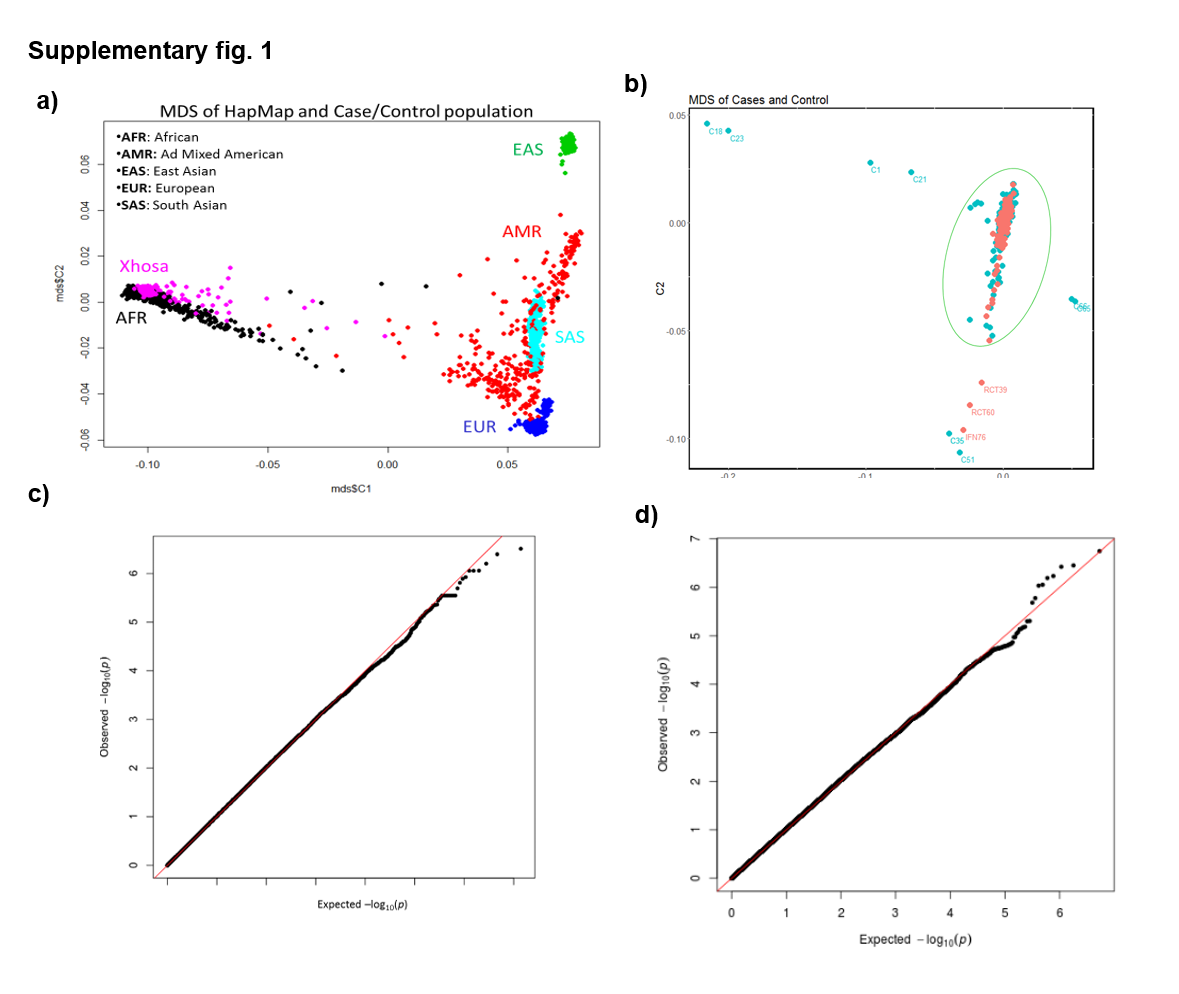


MDS of Discovery Cohort population. a) Genome-wide SNP data were used to compared cases and controls from the South African discovery cohort to the HapMap samples: samples cluster together with the other AFR samples from the HapMap. b) Discovery cohort cases and controls were compared to identify samples that are genetic outliers. Case and control samples outside the elipse were removed from further analysis. c) Q-Q plot of the observed and expected quantiles of the GWAS p values. The observed p values follow the expected p value and diverge for small p values.


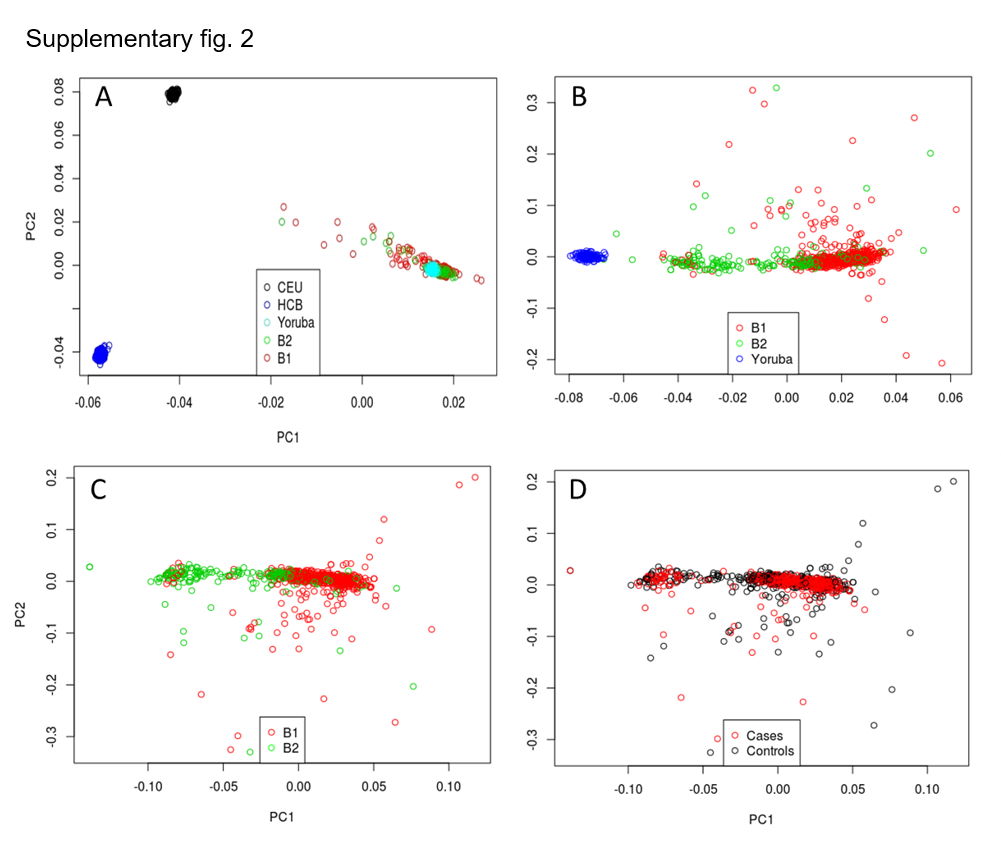
MDS of Combined cohort population compared to HapMap populations. a) PCA of HapMap populations; CEU, HCB and Yoruba; study series Discovery (B1) and Validation (B2). b) PCA of Yoruba and study cohorts B1 and B2 c) PCA of study cohorts B1 and B2 d) PCA of all study samples in B1 and B2, labelled by case/ control status.

Supplementary Table 1: List of top 50 differentially-expressed genes upon *C neoformans* stimulation

Supplementary Table 2: Gene Ontology terms associated with genes differentially expressed in PBMCs following 24h *C. neoformans* stimulation
