# Supplementary material for: Genome-Wide Association Study Identifies Novel Colony Stimulating Factor 1 Locus Conferring Susceptibility to Cryptococcosis in Human Immunodeficiency Virus-Infected South Africans
Source: Open Forum Infect Dis. 2020 Oct 16;7(11):ofaa489. doi: 10.1093/ofid/ofaa489 (PMC7686661; doi:10.1093/ofid/ofaa489)
Supplement: ofaa489_suppl_Supplementary_Materials [file ofaa489_suppl_supplementary_materials.docx]

**Online Methods**

1. **Genotyping and association analyses**

**Discovery cohort**

DNA from 524 samples was extracted using QIAamp DNA Blood Kit (QIAGEN) and genotyped using the Illumina HumanOmniExpressExome-8 v1.0 SNP chip. Genotype calling was performed using optiCall 0.7.0[1] using the default setting. Eleven genetically divergent samples identified in multi-dimensional scaling plots with HapMap data were excluded from further analysis. Relatedness analysis using PLINK was performed to remove closely related individuals. 245,091 variants that passed quality controls were aligned to the 1000 Genome reference using Genotype Harmonizer[2]. The data were phased using SHAPEIT2 v2.r644 algorithm[3] and imputed using the Michigan Imputation server[4] using the IMPUTE2 algorithm[5]. Post-imputation QC were used to remove low-quality (r2 <= 0.8) imputed variants before further analyses.

**Validation cohort**

DNA from 211 samples was extracted on the chemagic Prepito®-D instrument using the Prepito® DNA Blood kit (PerkinElmer, [Massachusetts](https://en.wikipedia.org/wiki/Waltham,_Massachusetts), USA). To remove any contaminating magnetic beads DNA samples were placed on a 96R Super Magnet Plate (**Alpaqua Engineering, LLC,** [Massachusetts](https://en.wikipedia.org/wiki/Waltham,_Massachusetts), USA) and DNA transferred. DNA quality and quantity was determined using a Nanodrop 2000c spectrophotometer (Thermo Scientific, [Massachusetts](https://en.wikipedia.org/wiki/Waltham,_Massachusetts), USA). Genotyping was performed using the Illumina GSA beadchip GSA MD v1 and genotype calling performed using Illumina GenomeStudio using the default setting. Relatedness analysis using PLINK was performed to remove any closely related individuals: 6 samples that failed quality control were removed from further analyses. Sample variants were aligned to the 1000 Genome reference using HRC checking tool (<https://www.well.ox.ac.uk/~wrayner/tools/>). The data were further phased to the phase 3 reference panel using SHAPEIT2 v2.r644 algorithm[6] and imputed using the Michigan Imputation server using Minimac4 ([https://imputationserver.sph.umich.edu/index.html#](https://imputationserver.sph.umich.edu/index.html)!). Post-imputation QC were used to remove low-quality (r2 <= 0.8) imputed variants before further analyses.

Although the HumanOmniExpress -8 chip has a greater number of markers, the GSA array was better suited to genotype our African Populations. On the HumanOmniExpress -8 chip the proportion of genomic variants imputed with an LD r2 value at 0.8, at MAF>5% in the reference HapMap AFR (African) population, is 0.43. In contrast, for the GSA array the proportion of genomic variants imputed with an LD r2 value of 0.8, is much higher at 0.8.

**Association analysis**

Population stratification that might influence the association test was accessed using MDS analysis (Supplementary Fig. 2) implemented in the GCTA package ([https://cnsgenomics.com/software/gcta/#Overview](https://eur03.safelinks.protection.outlook.com/?url=https%3A%2F%2Fcnsgenomics.com%2Fsoftware%2Fgcta%2F%23Overview&data=01%7C01%7Cshichina.kannambath%40kcl.ac.uk%7Cc509a1975de04844411708d71726eef6%7C8370cf1416f34c16b83c724071654356%7C0&sdata=Eg5HmqT7ghKCurB6ITT8%2F5ngyyG7jZeZqCfgtKGEUPw%3D&reserved=0)); we included 15 principal components as covariates in the association analysis. The association analysis was performed using PLINK 1.9 ([http://pngu.mgh.harvard.edu/~purcell/plink](http://pngu.mgh.harvard.edu/%7Epurcell/plink)). Genetic susceptibility to disseminated cryptococcosis was tested using logistic regression. P value distribution was assessed by a Quantile-Quantile (Q-Q) plot and there was no inflation effect on the association analysis (Fig. 3). Imputed datasets for each series were then merged into one prior to association analysis. The association analysis was performed using GEMMA v0.98.1 ([https://github.com/genetics-statistics/GEMMA](https://eur03.safelinks.protection.outlook.com/?url=https%3A%2F%2Fgithub.com%2Fgenetics-statistics%2FGEMMA&data=01%7C01%7Cshichina.kannambath%40kcl.ac.uk%7Cc509a1975de04844411708d71726eef6%7C8370cf1416f34c16b83c724071654356%7C0&sdata=KHHNqftOFA5uSq7cRmDl55ifQYsOZUm7VNB4sCa8TLs%3D&reserved=0)) on 2,686,126 variants. QQ plot and Manhattan plots were generated using QQman[7]. p < 5x10^-6^ was considered the threshold for significant association.

***Code availability*:** All our stepwise methods and analysis scripts are available online ([https://github.com/alanmichaelpittman100/Crypto-GWAS](https://eur03.safelinks.protection.outlook.com/?url=https%3A%2F%2Fgithub.com%2Falanmichaelpittman100%2FCrypto-GWAS&data=01%7C01%7Cshichina.kannambath%40kcl.ac.uk%7Cc509a1975de04844411708d71726eef6%7C8370cf1416f34c16b83c724071654356%7C0&sdata=4%2FUl6%2Bf%2FhOq8iOPXYq68r398JBHrmF%2BbzSwOzSYdA0g%3D&reserved=0)).

**Analysis of impact of non-coding variants on gene expression**. Single nucleotide polymorphisms with significant p values were further analysed. To explore any possible impact of candidate non-coding variants/ possible regulatory SNPs on gene expression, information on SNP association with annotated genes was collected using the online tool HaploReg (http://www.broadinstitute.org/ mammals/haploreg/haploreg.php v4.1[8]). Information on variants within 500kb of each SNP was collated. Genotype Tissue Express eQTL data for significant SNPs were analysed using the HaploReg database and GTEx Portal (<https://gtexportal.org/home/> accessed Sept 1^st^, 2018; Release V7). HaploReg collects eQTL and LD information from large genomics projects such as ENCODE, the 1000 Genome Project, GTEx (NIH RNA-seq project on multiple tissues from cadavers) and GEUVADIS (EU RNA-seq project). We checked for existing eQTL information for all the top SNPs identified (Table 2). The effect of the CSF1 gene enhancer region containing rs12124202 was analysed using various databases for gene enhancer and ChIPseq data (<https://www.encodeproject.org/>, <https://www.genecards.org/>, <https://genome.ucsc.edu/cgi-bin/hgTrackUi?db=hg19&g=geneHancer>).**Pathway enrichment and gene ontology analyses** of genes associated with p<5x10^-3^ were performed using DAVID 6.8 (<https://david.ncifcrf.gov/>).

**Meta-analysis** of counts of the genotyped SNP rs1999713 at the CSF1 locus, which was hard-called (non-imputed) on both genotyping platforms and present in both cohorts, was performed for the combined discovery and replication cohorts to negate any uncertainty from imputation, using an allele and fixed effects model in metagenyo software[9].

1. **PBMC isolation and culture**

PBMCs were isolated from control and patient blood samples according to a standard Ficoll-Paque plus (GE healthcare) protocol. Briefly, the PBMC fraction was obtained by laying diluted blood samples over Ficoll-Paque plus and centrifuging at 450g for 30min. The PBMC fraction was collected and washed twice in phosphate buffered saline (PBS). PBMCs were then counted with the cell counter (Bio-Rad) and cell number was adjusted to 5x10^5^ cells/ml. PBMCs were cultured in RPMI 1640 media supplemented with gentamicin 10 mg/mL, L-glutamine 10 mM, pyruvate 10 mM and 10% human serum (Sigma, UK).

1. **Fungal strains and culture**

*Cryptococcus neoformans* (*Cn*, serotype A reference strain H99) was cultured on Sabouraud dextrose (SD) agar and incubated for 48h at 30^0^C. Up to 5 *Cn* colonies were sampled and inoculated in 5ml of SD liquid media and cultured overnight at 30^0^C with gentle agitation (165 rpm). Fungal cells were collected after centrifugation (200 rpm for 5min) and washed twice in PBS. Heat killed (HK) *Cn* were prepared by incubating the fungal cells at 65^0^C for 2h. Cells were washed twice in PBS prior to opsonising with monoclonal anti-capsule (18B7 [10]) antibody. For the phagocytosis assay, opsonised *Cn* were stained with Calcofluor White (Sigma) for 15min prior to co-culture with PBMCs.

1. **M-CSF functional characterisation experiments**

For the fungal killing assay, 1x10^6^ PBMCs from HIV-infected patients (n=5) and healthy volunteers were pre-treated with 100 ng/ml M-CSF (R&D Systems) or 15 ng/ml anti-MCSF (AF216-SP, R&D Systems) antibody. Cells were incubated in a flat-bottom 96-well plate for 24h at 37^0^C. PBMCs were then challenged with HK Cn (MOI=0.1). After 24h of co-culture, plates were briefly centrifuged, and the supernatant was carefully removed. Host cells were then lysed with 100 µl of water for 10 min. Samples were collected from each well and 1:10000 dilution was plated onto fresh SAB agar. Plates were incubated at 30^0^C for 48h and Colony forming units (CFU) counted. Fungal killing was defined as the percentage of cryptococcal growth relative to the input inoculum (1x10^5^) and calculated as ([CFU in experimental well/inoculum]) × 100 as described previously[11].

For the phagocytosis assays, PBMCs were pre-treated and cultured as above. After 24h, PBMCs were challenged with calcoflour white pre-stained opsonised HK *Cn* (MOI=0.1) for 24h at 37^0^C. Cells were harvested with ice-cold PBS and washed twice in PBS. PBMCs were then stained with nuclear stain DRAQ5 (Cell signalling) and antiCD14-PE (Clone  MφP9, BD Bioscience) and then fixed with fixation buffer (BioLegend). Cells were then run on the BD FACSCANTO flow cytometer (BD Biosciences) and analysed using Flow Jo software (Oregon, US). For the analysis, doublets were removed using the forward scatter gating and the monocytes were selected as CD14^+ve^ and DRAQ5^+ve^. Phagocytosis was identified as cells with CD14-PE^+ve^ and calcofluor white^+ve^. Percentage of cells with phagocytosis was identified from the total number of CD14^+ve^ monocytes. Experiments were repeated 4-8 times dependent on PBMC availability.

1. **RNA sequencing and analysis**

PBMCs were isolated from the healthy Xhosa volunteers as described above. PBMCs (5x10^5^) were stimulated with heat-killed Cn (MOI 0.1) for 24h. RNA was extracted from unstimulated and stimulated PBMCs using TRIzol® (Life technologies) reagent according to the manufacturer’s instructions. RNA was further purified using an RNAeasy kit (QIAGEN) with an additional purification step by on-column DNase treatment using the RNase-free DNase Kit (QIAGEN) to ensure elimination of genomic DNA. RNA quality was analysed using a 2200 Tape station (Agilent Technologies) and RNA with a RIN greater than 8.0 was used for library preparation. RNA-seq libraries were prepared using the mRNA seq kit v2 (Illumina), according to manufacturer’s instructions. The libraries were size selected using AMPure XP beads (Beckman Coulter), assessed using a 2200 Tape station and quantified using a KAPA Illumina SYBR Universal Lib QPCR kit (Anachem Ltd, Bedfordshire, UK) and broad range Qubit analysis using the QuantiT dsDNA BR assay (Life technologies). Libraries were then sequenced to generate 150-bp paired-end reads on an Illumina HiSeq 2500 (Imperial College London). Read quality was assessed using FASTQC (<https://www.bioinformatics.babraham.ac.uk/projects/fastqc/>) and reads mapped to the human reference genome (hg19) using the read alignment software TopHat2 with default parameters[12]. Duplicate reads were removed using Picard software (http://broadinstitute.github.io/picard/index.html). The reads were annotated and gene counts obtained using HTSeq[13]. Differentially expressed (DE) genes between controls and Cn-treated samples were identified using DEseq2[14] . Gene ontology and pathway analyses were performed using DAVID[15] for these DE genes.

References

1. Shah TS, Liu JZ, Floyd JAB, et al. OptiCall: A robust genotype-calling algorithm for rare, low-frequency and common variants. Bioinformatics **2012**; 28:1598–1603.

2. Deelen P, Bonder MJ, Van Der Velde KJ, et al. Genotype harmonizer: Automatic strand alignment and format conversion for genotype data integration. BMC Res Notes **2014**; 7.

3. Delaneau O, Zagury JF, Marchini J. Improved whole-chromosome phasing for disease and population genetic studies. Nat. Methods. 2013; 10:5–6.

4. Das S, Forer L, Schönherr S, et al. Next-generation genotype imputation service and methods. Nat Genet **2016**; 48:1284–1287.

5. Howie B, Marchini J, Stephens M. Genotype Imputation with Thousands of Genomes. G3&amp;#58; Genes|Genomes|Genetics **2011**; 1:457–470.

6. Delaneau O, Howie B, Cox AJ, Zagury JF, Marchini J. Haplotype estimation using sequencing reads. Am J Hum Genet **2013**;

7. D. Turner S. qqman: an R package for visualizing GWAS results using Q-Q and manhattan plots. J Open Source Softw **2018**;

8. Ward LD, Kellis M. HaploReg: A resource for exploring chromatin states, conservation, and regulatory motif alterations within sets of genetically linked variants. Nucleic Acids Res **2012**; 40.

9. Martorell-Marugan J, Toro-Dominguez D, Alarcon-Riquelme ME, Carmona-Saez P. MetaGenyo: A web tool for meta-analysis of genetic association studies. BMC Bioinformatics **2017**;

10. Macura N, Zhang T, Casadevall A. Dependence of macrophage phagocytic efficacy on antibody concentration. Infect Immun **2007**; 75:1904–1915.

11. Brouwer AE, Rajanuwong A, Chierakul W, et al. Combination antifungal therapies for HIV-associated cryptococcal meningitis: A randomised trial. Lancet **2004**; 363:1764–1767.

12. Kim D, Pertea G, Trapnell C, Pimentel H, Kelley R, Salzberg SL. TopHat2: accurate alignment of transcriptomes in the presence of insertions, deletions and gene fusions. Genome Biol **2013**; 14:R36.

13. Anders S, Pyl PT, Huber W. HTSeq – A Python framework to work with high-throughput sequencing data HTSeq – A Python framework to work with high-throughput sequencing data. Bioinformatics **2014**; 31:0–5.

14. Love MI, Huber W, Anders S. Moderated estimation of fold change and dispersion for RNA-seq data with DESeq2. Genome Biol **2014**; 15.

15. Huang DW, Sherman BT, Lempicki RA. Systematic and integrative analysis of large gene lists using DAVID bioinformatics resources. Nat Protoc **2009**; 4:44–57.
